# Impact of Ascending HPV Infection on Colorectal Cancer Risk: Evidence from a Nationwide Study

**DOI:** 10.3390/microorganisms12091746

**Published:** 2024-08-23

**Authors:** Pin-Ho Pan, Ci-Wen Luo, Wen-Chien Ting, Bei-Hao Shiu, Jing-Yang Huang, Stella Chin-Shaw Tsai, Frank Cheau-Feng Lin

**Affiliations:** 1 Division of Pediatric Gastroenterology, Department of Pediatrics, Tungs’ Taichung MetroHarbor Hospital, Taichung 43503, Taiwan; t6395@ms3.sltung.com.tw; 2 Department of Post-Baccalaureate Medicine, National Chung Hsing University, Taichung 402202, Taiwan; 3 Department of Medical Research, Tungs’ Taichung MetroHarbor Hospital, Taichung 43503, Taiwan; t14825@ms3.sltung.com.tw; 4 School of Medicine, Chung Shan Medical University, Taichung 40201, Taiwan; cshy1294@csh.org.tw; 5 Division of Colorectal Surgery, Department of Surgery, Chung Shan Medical University Hospital, Taichung 40201, Taiwan; shiubeihao@gmail.com; 6 Institute of Medicine, Chung Shan Medical University, Taichung 40201, Taiwan; cshe961@csh.org.tw; 7 Department of Medical Research, Chung Shan Medical University Hospital, Taichung 40201, Taiwan; 8 Superintendent Office, Tungs’ Taichung MetroHarbor Hospital, Taichung 43503, Taiwan; 9 College of Life Sciences, National Chung Hsing University, Taichung 402202, Taiwan; 10 Department of Surgery, Chung Shan Medical University Hospital, Taichung 40201, Taiwan

**Keywords:** human papillomavirus (HPV), colorectal cancer, anal cancer, risk factors, Cox regression analysis

## Abstract

Colorectal cancer (CRC) is a prevalent and escalating health issue in Taiwan. This nationwide study delves into the relationship between Human Papillomavirus (HPV) infection and CRC risk, employing population datasets from 2007 to 2017. Cox regression analyses revealed a statistically significant hazard ratio (HR) of 1.73 (95% CI: 1.63–1.83) for CRC in HPV-positive patients, indicating a considerably elevated risk compared to non-infected individuals. Further, stratification by sex showed males with HPV have a higher CRC risk (HR = 1.49, 95% CI: 1.40–1.58) compared to females. Age-related analysis uncovered a progressive increase in CRC risk with advancing age (HR = 34.69 for over 80 years). The study of specific CRC subtypes showed varying risks: HR = 1.74 for the colon, HR = 1.64 for the rectum, and a notably higher HR = 4.72 for the anus. Comorbid conditions such as hypertension (HR = 1.26), diabetes mellitus (HR = 1.32), and abnormal liver function (HR = 1.18) also correlate with significantly increased CRC risks. These findings suggest that HPV is a significant risk factor for CRC, with disparities in risk based on anatomical location, demographic characteristics, and comorbidities, highlighting the need for intervention strategies and targeted prevention.

## 1. Introduction

Human papillomavirus (HPV) is widely recognized as a causative agent for several cancers, most notably cervical cancer [1]. Recent evidence, however, points to its potential role in the pathogenesis of colorectal cancer (CRC) [2,3]. CRC is one of the leading causes of cancer-related mortality worldwide, with significant incidence rates in both developed and developing countries [4]. The rising incidence of CRC has led to increased healthcare costs, posing a substantial burden on medical systems globally [4,5]. In North America and Europe, studies have explored the potential link between HPV infection and CRC [6,7]. These studies have shown varying degrees of association, suggesting that HPV could be a contributing factor in colorectal carcinogenesis. For instance, research has identified HPV DNA in colorectal tissue samples, indicating a possible viral involvement in the disease process [8,9]. Furthermore, the presence of HPV-induced inflammatory cytokines such as interleukin-6 (IL-6) and interleukin-8 (IL-8) has been implicated in promoting a pro-inflammatory environment conducive to cancer development [10,11].

Animal studies further corroborate the hypothesis that HPV could influence CRC. These studies have demonstrated that HPV infection leads to chronic inflammation, a recognized risk factor for colorectal cancer. Specifically, HPV oncogenes have been shown to upregulate pro-inflammatory cytokines such as IL-6 and tumor necrosis factor-alpha (TNF-α) [12,13], both key players in inflammation and associated with CRC progression [14]. Moreover, IL-12p70, another cytokine linked with immune response regulation, has been found to correlate with risk, suggesting a complex interplay between viral infection and immune modulation [15,16]. In Asia, where CRC incidence rates vary significantly across regions, exploring the epidemiological relationship between HPV and CRC is particularly pertinent [16,17,18]. Studies conducted in Taiwan, a country with a high incidence of CRC and a well-established healthcare system, offer valuable insights into this association within an Asian population [2]. Understanding the specific risk factors and underlying mechanisms contributing to HPV-associated CRC in this context can inform targeted interventions and public health strategies to mitigate the burden of the disease [19,20].

Despite these findings, the interplay between HPV infection and other risk factors for CRC, such as comorbid conditions, remains less understood. This study also considers the role of comorbidities such as hypertension, diabetes, and obesity in modulating CRC risk among HPV-positive and HPV-negative individuals. These comorbid conditions have been shown to have varying prevalence between HPV-positive and HPV-negative cases, potentially influencing CRC risk through distinct oncogenic pathways. Similar to the dual pathways observed in vulvar cancer—one related to HPV and another to chronic lesions like lichen sclerosus—it is plausible that our results suggest different pathways for CRC development depending on HPV status and associated comorbidities [21,22,23].

Given the accumulating evidence of a significant epidemiological and mechanistic link between HPV infection and CRC across various populations, it is imperative to understand this relationship in depth. This understanding is crucial for the development of targeted prevention and treatment strategies that could significantly reduce the healthcare burden associated with CRC. The present study, therefore, aims to investigate the risk relationship between HPV infection and CRC using data from the Taiwan National Health Insurance Research Database. This research could offer foundational insights into modifying public health approaches to manage and prevent CRC in populations at risk.

## 2. Materials and Methods

### 2.1. Database Sources

The Taiwan National Health Insurance Research Database (NHIRD) stands as an exemplary repository of population-level data. Initiated in 1995, the National Health Insurance (NHI) program operates as a single-payer system aimed at enhancing healthcare accessibility and affordability. The NHIRD includes data from primary outpatient and inpatient settings since 2000, maintained by the Data Science Center of the Ministry of Health and Welfare (MOHW) since a 2016 update. Approved research datasets included sampling datasets with 2 million subjects, disease-specific databases, and complete population datasets. These de-identified datasets contain demographic information, disease diagnoses, prescriptions, operations, and investigations and can be linked to other research datasets, offering a powerful resource for biomedical research [24].

The Taiwan Cancer Registry (TCR), integrated within the Health and Welfare Data Center (HWDC), is an extensive national population registry dedicated to systematically collecting cancer-related data. Established in 1979, the TCR provides essential data on cancer incidence, treatment, and survival rates in Taiwan. The registry upholds high-quality data classified under the International Classification of Diseases, Ninth Revision, Clinical Modification (ICD-9-CM) before 2001, and the Tenth Revision (ICD-10-CM) from 2002 onwards. The HWDC manages de-identified patient data from various health databases, including the TCR, ensuring stringent data protection. Researchers must establish a remote connection to MOHW servers for on-site analysis, complying with strict privacy measures. The TCR, alongside the NHIRD, provides an invaluable resource for population-based cancer research and epidemiological studies [25].

### 2.2. Study Population

This study utilized medical records from the NHIRD database, covering 26 million Taiwanese individuals between 2007 and 2015, to gather data on HPV patients. HPV diagnoses were identified using ICD-9-CM codes 078.10, 078.19, 078.1x, 078.11, and 079.4x. From this dataset, 1,103,771 individuals diagnosed with HPV and 25,462,267 without HPV were identified [26,27]. We excluded individuals with an HPV history prior to 2007 and those with a cancer diagnosis before their HPV diagnosis. After applying 1:2 age and gender matching, the final study population included 939,874 individuals in the HPV group and 1,879,748 in the non-HPV group. Cases of cancer were identified based on diagnoses occurring after the index date, using ICD-10-CM codes C18, C19, C20, and C21. The classification of the primary site and histology of malignant tumors was conducted according to the International Classification of Diseases for Oncology, Third Edition (ICD-O-3), published by the World Health Organization in 2000. The relevant histology codes included 8041, 8144, 8210-11, 8255-8263, 8480-8481, 8490, 8510, 8570-8574, 6900, 6999, and 8000-8020, while excluding carcinoids (8240, 8249), neuroendocrine carcinomas (8246), and gastrointestinal stromal tumors (8936) [28]. Cases exhibiting malignant behavior codes were included in the Taiwan Cancer Registry (covering 1979–2015 data). Cases were classified into three categories: large intestine (C18, C19), rectum (C20), and anus (C21) [29].

### 2.3. Urbanization Classification

The urbanization level of the community was stratified into seven classifications, with level 1 representing the most urbanized areas and level 7 representing the least urbanized areas. This classification system was introduced by Liu and colleagues at the Taiwan National Health Research Institute, based on data from the 2000 Taiwan census. The criteria for this classification include population density, the proportion of residents with a college education, the proportion of residents aged over 65, the ratio of physicians per 100,000 people, and other factors indicative of urbanization and socio-economic status [30].

### 2.4. Comorbidities

This study incorporated the following comorbidities as covariates in the regression analysis to reduce potential confounding effects [31,32]: ischemic heart disease (ICD-9: 410–414), hypertension (ICD-9: 401–405), stroke (ICD-9: 430–438), diabetes mellitus (ICD-9: 250), abnormal liver function (ICD-9: 794.8), renal failure (ICD-9: 584–586), gastrointestinal bleeding (ICD-9: 578), hyperlipidemia (ICD-9: 272), chronic kidney diseases (ICD-9: 585), chronic obstructive pulmonary disease (ICD-9: 490–496), peptic ulcer disease (ICD-9: 531–534), and gout (ICD-9: 274). This study incorporated comorbidities such as hypertension, diabetes mellitus, and obesity as covariates in the regression analysis to reduce potential confounding effects and better understand the differential impact on CRC risk between HPV-positive and HPV-negative individuals. These comorbidities were selected based on their known associations with both HPV infection and CRC risk [21,22,23,31,33]

### 2.5. Statistical Analysis

To improve comparability and reliability, we accounted for known confounding factors and adjusted for multiple variables to ensure the accuracy of our results. The chi-square test was utilized to compare categorical variables such as sex, income, and urbanization level between HPV and non-HPV patients. We conducted the Shapiro-Wilk test to evaluate the normality of the distribution (*p* < 0.05). The Wilcoxon rank-sum test was used to analyze differences in continuous variables between the groups. For case-control sampling, we used multivariate stratified Cox regression models to estimate the hazard ratio (HR) and 95% confidence interval (CI) for secondary outcomes. To support inference, we provided straightforward estimation equations and proposed a more efficient method. This framework validated the inclusion of status as a covariate in the regression models. A multivariate Cox regression model was employed to assess the risk for each participant, with results expressed as HR and 95% CI. Data analysis was performed using SAS 9.4 software (SAS Institute, Cary, NC, USA), and statistical significance was set at *p* < 0.05.

## 3. Results

### 3.1. Baseline Characteristics

Table 1 outlines the demographic characteristics of the study population and the differences between participants with and without HPV. Due to matching, gender and age did not show statistically significant differences. However, urbanization level revealed notable distinctions: the majority of HPV patients (34.01%) lived in the highest urbanization areas, whereas most non-HPV patients (31.23%) resided in the second-highest urbanization areas, showing a statistically significant difference. Regarding residential areas, both groups had the highest numbers in the Taipei region, but HPV patients (42.10%) had a significantly higher proportion compared to non-HPV patients (35.72%). In terms of insurance coverage, most HPV patients were covered by Labor insurance (65.84%), followed by Unemployment insurance (13.00%). Similarly, non-HPV patients were mainly covered by Labor insurance (62.94%), followed by Unemployment insurance (15.66%), which also displayed a statistically significant difference. Among comorbidities, no significant differences were found in stroke and abnormal liver function. However, diabetes mellitus was more prevalent among non-HPV patients (5.14%) than HPV patients (4.98%). HPV patients exhibited higher proportions in other comorbidities, all showing statistically significant differences.

### 3.2. Risk Relationship between HPV and Cumulative Incidence of CRC

**Figure 1:**
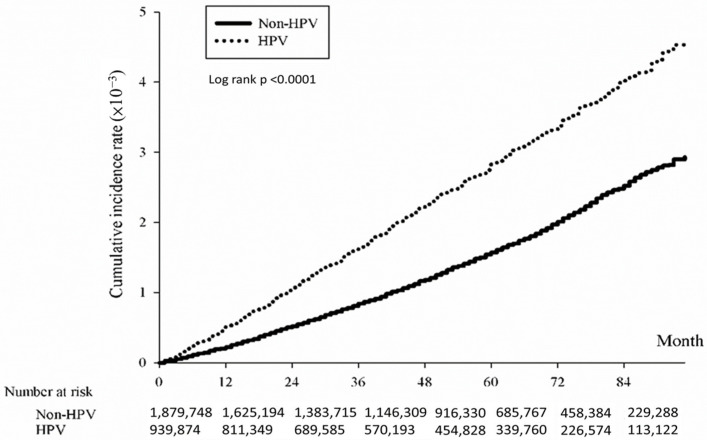
Kaplan-Meier curve illustrating cumulative incidence of colorectal cancer in patients with/without HPV.

**Figure 2:**
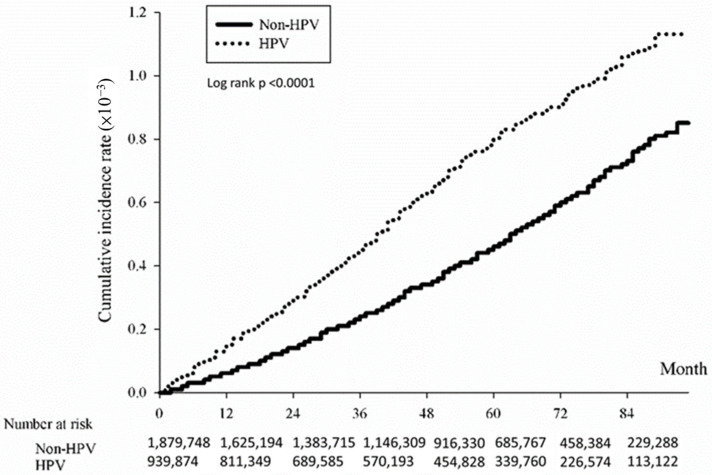
Kaplan-Meier curve illustrating cumulative incidence of colorectal cancer in the colon subtype of patients with/without HPV.

**Figure 3:**
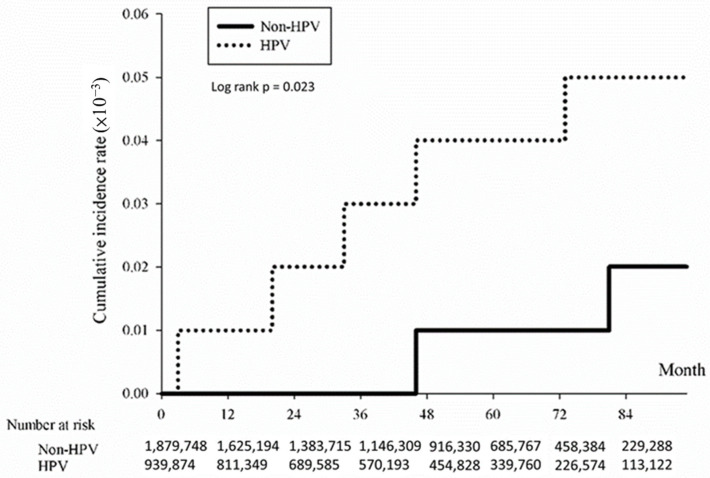
Kaplan-Meier curve illustrating cumulative incidence of colorectal cancer in the rectum subtype of patients with/without HPV.

**Figure 4:**
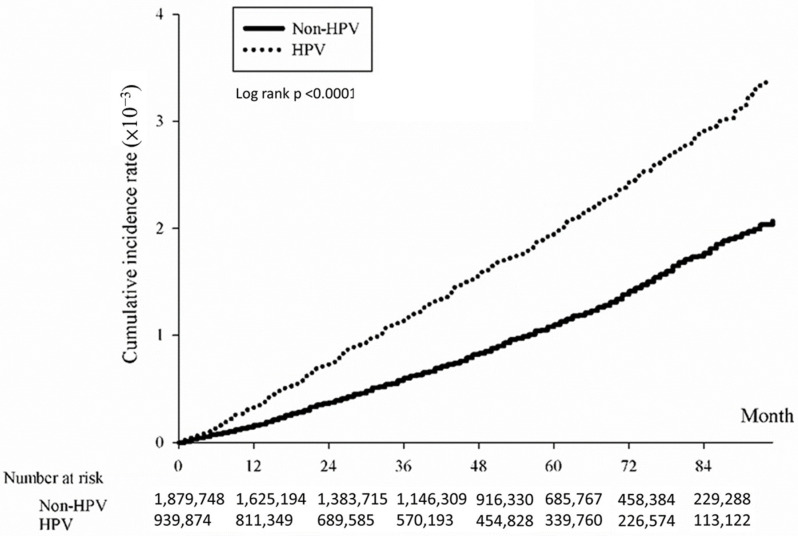
Kaplan-Meier curve illustrating cumulative incidence of colorectal cancer in the anus subtype of patients with/without HPV.

The association between HPV infection and the cumulative incidence of CRC was evaluated using the Kaplan-Meier method. Results indicated a significantly heightened risk of developing CRC over time among individuals with HPV compared to those without HPV. The study determined an overall hazard ratio for CRC associated with HPV of 1.74 (*p* < 0.0001), indicating a significantly elevated risk within the HPV-positive group (Figure 1). Subtype analysis revealed significant associations with CRC subtypes: colon (Figure 2; HR = 1.77, *p* < 0.0001), rectum (Figure 3; HR = 1.62, *p* < 0.0001), and anus (Figure 4; HR = 4.86, *p* < 0.0001).

### 3.3. Cox Regression

Table 2 presents the risk assessment of HPV on colorectal cancer (CRC) after adjusting for sex, age, urbanization, area, insurance coverage, and comorbidities. The analysis revealed a statistically significant hazard ratio (HR) of 1.73 (95% CI: 1.63–1.83) for HPV-associated CRC, indicating a higher risk among patients with HPV. Males exhibited a higher risk of CRC compared to females, with an HR of 1.49 (95% CI: 1.40–1.58). Regarding age, there was a significant increase in CRC risk with advancing age: for the age group under 20 years, HR = 0.04 (95% CI: 0.02–0.08); for 40–60 years, HR = 6.81 (95% CI: 6.00–7.72); for 60–80 years, HR = 18.67 (95% CI: 18.67–24.20); and for over 80 years, HR = 34.69 (95% CI: 29.78–40.40). Urbanization level 6 exhibited a lower risk of CRC compared to the highest level, with an HR of 0.71 (95% CI: 0.58–0.85). Among the regions, only Southern Taiwan showed a higher risk of CRC than Taipei, with an HR of 1.20 (95% CI: 1.09–1.33). No statistically significant difference was observed based on insurance coverage. However, hypertension (HR = 1.26, 95% CI: 1.12–1.42), diabetes mellitus (HR = 1.32, 95% CI: 1.22–1.43), and abnormal liver function (HR = 1.18, 95% CI: 1.07–1.30) were associated with a higher risk of CRC, all statistically significant.

In Table 3, we further examined the relationship between HPV and various types of CRC using multivariate Cox regression, adjusting for sex, age, urbanization, area, insurance coverage, and comorbidities. The results revealed adjusted hazard ratios (HR) for HPV-associated subtypes: for the colon, the adjusted HR was 1.74 (95% CI: 1.62–1.87); for the rectum, the adjusted HR was 1.64 (95% CI: 1.47–1.84); and for anus, the adjusted HR was 4.72 (95% CI: 2.39–9.31), all of which were statistically significant. Notably, the adjusted HR for HPV-associated anus was the highest among the subtypes examined.

## 4. Discussion

In this study, we examined the association between HPV and the risk of developing various types of CRC using multiple Cox regression models, adjusting for key variables such as sex, age, urbanization level, residence area, insurance coverage, and comorbidities. The findings consistently revealed a significantly elevated hazard ratio (HR) for CRC among individuals with HPV across all interfering factors and risk factors analyzed. Specifically, HPV was linked to an increased risk for cancers of the colon, rectum, and anus, with the highest HR observed for anal cancer. These results accentuate the strong association between HPV and a heightened risk of CRC, highlighting the importance of targeted interventions and the need for further research into preventive strategies.

The higher incidence of anal cancer in Southern Taiwan, as observed in our study, may be influenced by the significant prevalence of HIV infection and tobacco use in this region. A recent study reported that AIDS incidence increased from 14.9% to 31.2% in the Southern area and Kaoping area, highlighting the substantial burden of HIV in this region [34]. HIV-infected individuals are at a heightened risk of HPV-related malignancies due to their immunosuppressed state, which facilitates persistent HPV infections and subsequent cancer progression. Furthermore, tobacco use is a well-established risk factor that exacerbates the carcinogenic potential of HPV. The combined impact of HIV and tobacco use may contribute to the observed higher incidence of anal cancer and potentially influence its progression to colorectal cancer. Future research should focus on the interplay between these factors to develop targeted prevention and intervention strategies.

Our findings suggest that the increased risk of CRC in HPV-positive patients may be influenced by the presence of comorbid conditions such as hypertension, diabetes, and obesity. These comorbidities might contribute to the carcinogenesis process through chronic inflammation and metabolic dysregulation. This is similar to the dual pathways observed in vulvar cancer, where HPV-related oncogenic pathways coexist with pathways related to chronic inflammatory conditions. The higher prevalence of certain comorbidities among HPV-positive individuals indicates the possibility of different oncogenic pathways leading to CRC, emphasizing the need for targeted prevention and treatment strategies that consider both viral and metabolic factors [21,22,23].

Moreover, the interplay between HPV infection and these comorbidities underscores the complexity of CRC pathogenesis. For instance, hypertension and diabetes are known to contribute to a pro-inflammatory state, which may exacerbate the oncogenic potential of HPV by promoting a microenvironment conducive to cancer development [22,35,36,37]. Similarly, obesity, through its association with chronic inflammation and metabolic alterations, may further increase the risk of CRC in HPV-positive individuals [38,39].

Furthermore, sexual behavior is a significant factor influencing the transmission and persistence of HPV, thereby impacting the risk of HPV-related cancers. Behaviors such as having multiple sexual partners, frequent sexual activity, and inconsistent use of protection increase the likelihood of HPV transmission [40]. Persistent HPV infections, particularly with high-risk oncogenic types such as HPV 16 and 18, are closely linked to the development of cancers in the anogenital region, including cervical, anal, and penile cancers [7,38].

The higher incidence of anal cancer observed in certain populations, such as men who have sex with men and HIV-infected individuals, underscores the role of sexual behavior in HPV transmission and cancer risk [7]. In HIV-infected individuals, the immunosuppressed state facilitates the persistence of HPV infections and enhances their oncogenic potential [38]. Understanding the interplay between sexual behavior, HPV transmission, and cancer development is crucial for developing targeted prevention and intervention strategies.

Elucidating these dual pathways is crucial for developing more effective prevention and treatment strategies. Targeted interventions that address both HPV infection and the management of comorbid conditions could potentially reduce the overall burden of CRC. Future research should focus on elucidating the precise mechanisms through which these comorbidities interact with HPV to influence CRC risk, as well as on developing integrated public health strategies to mitigate these risks

The concept of retrograde infection for HPV, particularly in the colorectal region, suggests that HPV can ascend from the anal area to the colon, despite the primarily downward processes of peristalsis and defecation. Several mechanisms have been proposed to explain this phenomenon. Firstly, HPV can infect mucosal surfaces and spread locally through the mucosa from the anus to the rectum and potentially to the colon. This local spread can occur particularly in the presence of microabrasions or tears that facilitate viral entry and movement [41,42]. Additionally, HPV-infected cells can evade the immune system and migrate along epithelial surfaces. This migration allows infected epithelial cells to move and proliferate, leading to the potential spread of the virus from the initial site of infection to adjacent areas [43,44]. Another key mechanism involves the inflammatory response induced by HPV infection, which can disrupt normal tissue architecture and barriers, allowing the virus to spread. Chronic inflammation creates an environment conducive to the movement of infected cells and viruses along the mucosal surfaces [38,39]. Furthermore, lymphatic spread is another potential pathway, although less common. The lymphatic system drains various tissues, and infected lymphatic fluid could carry the virus from the anal region to other parts of the lower gastrointestinal tract [45,46]. These mechanisms collectively suggest that while the primary direction of gastrointestinal content movement is downward, HPV can utilize specific biological pathways to move in a retrograde manner, leading to infections in the rectum and colon [47,48].

Understanding the genetic and molecular basis of HPV-induced carcinogenesis in colorectal tissues is crucial for elucidating the pathways involved in retrograde infection. HPV’s oncoproteins, E6 and E7, play pivotal roles in disrupting normal cell cycle control. E6 promotes the degradation of the tumor suppressor protein p53, preventing apoptosis, while E7 binds to and inactivates the retinoblastoma protein (pRb), leading to uncontrolled cell proliferation and genomic instability [45,49]. This disruption allows for the accumulation of mutations and chromosomal aberrations that are hallmarks of cancer [50]. The pro-inflammatory state induced by chronic HPV infection, characterized by the upregulation of cytokines such as interleukin-6 (IL-6) and tumor necrosis factor-alpha (TNF-α), further promotes oncogenesis by creating a microenvironment conducive to cancer development [51,52]. Additionally, HPV’s ability to evade the host immune system allows for prolonged infection and continued expression of viral oncoproteins, contributing to the persistence and progression of infection [53,54]. Advanced molecular techniques, such as polymerase chain reaction (PCR) and next-generation sequencing, are essential for detecting and quantifying HPV DNA in colorectal cancer tissues [55,56]. In vitro studies and animal models could provide valuable insights into the specific pathways and interactions involved in HPV-induced colorectal carcinogenesis [57,58]. By integrating these various approaches, researchers can gather comprehensive evidence to support the theory that HPV infection contributes to the development of colorectal and rectal cancers, ultimately informing potential preventive and therapeutic strategies [41].

Further examining the risk factors for CRC, we recognize the importance of behavioral and environmental influences on these outcomes [41]. Behavioral and environmental factors, such as urbanization and lifestyle, significantly influence CRC outcomes. Men generally have a higher incidence of CRC compared to women, partly due to differences in hormonal regulation. Estrogen plays a role in modulating ion transport in the colon and influencing hypoxia responses, which contribute to CRC development [59,60,61]. The risk of colorectal cancer (CRC) increases significantly with age, especially for those 80 and older, who have a hazard ratio of 34.69 [62]. This risk is due to age-related changes in sex steroids, receptor signaling, and a weakened immune response [63], including reduced T-cell numbers in older men [64]. The incidence of colorectal cancer (CRC) is higher in areas with greater urbanization compared to those with lower urbanization levels [65]. Behavioral and environmental factors, such as urbanization and lifestyle, significantly influence CRC outcomes [65,66,67,68,69,70,71]. Our findings reveal a trend where higher urbanization levels correlate with increased CRC risk.

One of our study’s notable findings is the higher risk of CRC in southern Taiwan. This is an intriguing phenomenon, given the differences in terrain, historical development, and cultural habits between the northern and southern regions [72]. CRC incidence is higher in urbanized areas due to lifestyle factors such as diet and physical activity levels. Urban living often leads to richer diets and less physical activity, increasing cancer risk. Higher urbanization levels also correlate with increased HPV prevalence, which is linked to greater interpersonal contact and higher sexually transmitted disease risks [73,74,75]. Hypertension, especially high SBP and DBP, is linked to increased colorectal cancer (CRC) risk, particularly in men [35,36]. ACE inhibitors and AT1R antagonists do not significantly impact tumor necrosis or survival in mice [76]. Type 2 diabetes (T2DM) also elevates CRC risk, with shared factors like obesity and inactivity [37,77]. Mice studies suggest diabetes affects CRC by inhibiting MAIT cells and altering the gut environment [78,79,80]. Higher liver markers (ALT, AST, GGT, TP, ALB) are linked to reduced CRC risk, and liver-derived metabolites like bile acids and bilirubin may influence CRC risk [81,82,83,84]. These findings emphasize the intricate relationship between metabolic and environmental factors in the development of colorectal cancer (CRC).

By understanding these mechanisms in greater detail, researchers can develop targeted interventions and preventive strategies to mitigate the risk of HPV-associated colorectal cancer. This comprehensive approach could significantly reduce the healthcare burden associated with CRC and improve patient outcomes.

This study is subject to certain limitations, including the absence of essential biochemical data about patients, as well as fundamental lifestyle factors such as exercise, smoking, alcohol consumption, and medication usage. Due to the inherent limitations posed by the National Health Insurance dataset used in our study, specific information on other lesions caused by HPV, such as cervical cancer, premalignant lesions, vulvar cancer, oropharyngeal cancer, and penile cancer, is not available. The dataset primarily focuses on colorectal cancer and does not contain detailed records of other HPV-related conditions. Additionally, the dataset does not include information on HPV genotyping, so we could not analyze the specific types of HPV, such as the highly oncogenic types 16 and 18. Furthermore, information on immunosuppressed patients or those treated with immunosuppressive treatments, including oral or parenteral corticosteroids, is not available in the dataset. Lastly, the dataset lacks detailed records of HPV vaccination status, preventing us from analyzing the number of vaccinated patients. Consequently, we were unable to include these data in our study. These factors were also considered outside the scope of our primary focus. Future research with access to more comprehensive datasets that include HPV genotyping, information on other HPV-related lesions, immunosuppression, and vaccination status could potentially address these gaps and provide a broader perspective on HPV-related malignancies.

These limitations notwithstanding, by employing matching techniques and making necessary adjustments, we have aimed to capture a representative snapshot of patients’ living conditions and health statuses, thus ensuring the accuracy of our results. Our study’s strength lies in its utilization of nationwide data on HPV patients from across Taiwan to examine the correlation with CRC. By focusing on CRC, the study provides valuable insights into the association between HPV infection and the risk of developing various types of CRC, highlighting significant epidemiological links and potential targets for intervention.

## 5. Conclusions

Our nationwide study establishes that HPV infection significantly elevates the risk of CRC. Analyzing data from Taiwan’s National Health Insurance Research Database over ten years, we found that HPV-positive individuals had a higher risk of CRC, with an HR of 1.73. Key findings include a higher risk for males (HR = 1.49) and the highest risk observed in individuals over 80 years old (HR = 34.69). Specifically, the HR for colon cancer was 1.59, and for rectal cancer, it was 1.68. Anal cancer exhibited the highest HR at 4.72. Notably, comorbid conditions such as hypertension (HR = 1.26), diabetes mellitus (HR = 1.32), and abnormal liver function (HR = 1.18) were associated with increased CRC risks.

These findings support the potential utility of expanding HPV vaccination programs to reduce CRC incidence. Although our study has limitations, including potential confounders like individual behavioral factors and genetic predispositions, it suggests that public health strategies might need revision to incorporate the broader implications of HPV infection, promoting a more comprehensive approach to cancer prevention.

## Figures and Tables

**Table 1:** Baseline characteristics of patients with/without HPV.

	Non-HPV	HPV	*p*
Sex			1.0000
Male	934,908 (49.74%)	467,454 (49.74%)	
Female	944,840 (50.26%)	472,420 (50.26%)	
Age			1.0000
0–19 years	513,000 (27.29%)	256,500 (27.29%)	
20–39 years	669,372 (35.61%)	334,686 (35.61%)	
40–59 years	469,150 (24.96%)	234,575 (24.96%)	
60–79 years	191,510 (10.19%)	95,755 (10.19%)	
80+ years	36,716 (1.95%)	18,358 (1.95%)	
Urbanization			<0.0001
1 (Highest level)	542,433 (28.86%)	319,694 (34.01%)	
2 (Very high level)	587,017 (31.23%)	290,511 (30.91%)	
3 (High level)	340,505 (18.11%)	162,859 (17.33%)	
4 (Moderately high level)	246,844 (13.13%)	109,824 (11.68%)	
5 (Moderate level)	36,687 (1.95%)	13,197 (1.40%)	
6 (Low level)	70,850 (3.77%)	23,620 (2.51%)	
7 (Lowest level)	55,412 (2.95%)	20,169 (2.15%)	
Area			<0.0001
Taipei	671,431 (35.72%)	395,713 (42.10%)	
Central	347,664 (18.50%)	180,479 (19.20%)	
Northern	279,894 (14.89%)	124,964 (13.30%)	
Kaohsiung, Pingtung	276,130 (14.69%)	115,919 (12.33%)	
Southern	261,434 (13.91%)	107,447 (11.43%)	
Eastern	43,195 (2.30%)	15,352 (1.63%)	
Insurance coverage			<0.0001
Labor insurance	1,183,084 (62.59%)	618,250 (65.62%)	
Unemployment	294,431 (15.66%)	122,189 (13.00%)	
Agricultural, irrigation, and fishery associations	91,295 (12.65%)	94,204 (10.00%)	
Low-income	73,295 (11.11%)	79,978 (8.84%)	
Public insurance	111,243 (9.39%)	79,000 (8.41%)	
Other	25,492 (1.79%)	17,858 (1.88%)	
Co-morbidity			
Hypertension	205,015 (10.91%)	112,709 (11.99%)	<0.0001
Hyperlipidemia	133,790 (7.12%)	83,114 (8.84%)	<0.0001
Peptic ulcer	122,250 (6.50%)	74,302 (7.91%)	<0.0001
Diabetes mellitus	96,584 (5.14%)	46,782 (4.98%)	<0.0001
Abnormal liver function	91,126 (4.85%)	45,661 (4.86%)	0.7009
Ischemic heart disease	63,872 (3.40%)	39,334 (4.19%)	<0.0001
Gout	49,184 (2.62%)	28,685 (3.05%)	<0.0001
Stroke	40,087 (2.13%)	20,246 (2.15%)	0.2386
COPD	32,326 (1.72%)	18,607 (1.98%)	<0.0001
Chronic kidney diseases	25,740 (1.37%)	14,747 (1.57%)	<0.0001
Renal failure	15,200 (0.81%)	8191 (0.87%)	<0.0001
GI bleeding	10,826 (0.58%)	5723 (0.61%)	0.0006

HPV, Human Papillomavirus; COPD, Chronic Obstruction Pulmonary Disease; GI, Gastrointestinal.

**Table 2:** Cox regression model of patients with/without HPV for colorectal cancer.

	Adjusted HR	95% CI	*p*
HPV	1.73	1.63–1.83	<0.0001
Sex			
Male	1.49	1.40–1.58	<0.0001
Female	Reference		
Age			
0–19 years	0.04	0.02–0.08	<0.0001
20–39 years	Reference		
40–59 years	6.81	6.00–7.72	<0.0001
60–79 years	21.26	18.67–24.20	<0.0001
80+ years	34.69	29.78–40.40	<0.0001
Urbanization			
1 (Highest level)	Reference		
2 (Very high level)	0.94	0.86–1.02	0.1157
3 (High level)	0.98	0.89–1.09	0.7578
4 (Moderately high level)	0.92	0.83–1.03	0.17
5 (Moderate level)	0.87	0.70–1.08	0.2077
6 (Low level)	0.71	0.58–0.85	0.0003
7 (Lowest level)	0.93	0.77–1.13	0.4672
Area			
Taipei	Reference		
Northern	0.95	0.85–1.06	0.3231
Central	1.03	0.94–1.13	0.5037
Southern	1.20	1.09–1.33	0.0004
Kaohsiung-Pingtung	1.03	0.93–1.13	0.6043
Eastern	0.98	0.78–1.22	0.833
Insurance coverage			
Public insurance	0.96	0.85–1.08	0.4893
Labor insurance	Reference		
Agricultural, Irrigation, Fishery Associations	1.09	0.99–1.20	0.0917
Low-income	1.19	0.85–1.67	0.3165
Unemployment	1.02	0.94–1.11	0.596
Other	0.97	0.75–1.26	0.8318
Co-morbidity			
Ischemic heart disease	0.99	0.91–1.08	0.8113
Hypertension	1.22	1.13–1.31	<0.0001
Stroke	1.06	0.95–1.18	0.3014
Diabetes mellitus	1.32	1.22–1.43	<0.0001
Abnormal liver function	1.18	1.07–1.30	0.001
Renal failure	1.13	0.88–1.45	0.3416
Gastrointestinal bleed	1.20	0.95–1.52	0.1367
Hyperlipidemia	1.07	0.99–1.16	0.0921
Chronic kidney diseases	1.22	1.00–1.50	0.0508
COPD	1.09	0.97–1.23	0.1389
Peptic ulcer	0.99	0.91–1.07	0.7521
Gout	1.01	0.91–1.13	0.827

Adjustment for sex, age, urbanization, area, insurance coverage, and co-morbidity. HPV, Human Papillomavirus; COPD, Chronic Obstruction Pulmonary Disease.

**Table 3:** Cox regression model of patients with/without HPV for the subtype of colorectal cancer.

Subtypes	Non-HPV		HPV			
	Event	Incidence Rate ^†^	Event	Incidence Rate ^†^	Crude HR	Adjusted HR
Colorectal cancer	2409	2.70 (2.60–2.81)	2083	4.70 (4.50–4.90)	1.739 (1.640–1.844)	1.727 (1.627–1.833)
Colon (C18, C19)	1694	1.90 (1.81–1.99)	1487	3.35 (3.19–3.53)	1.765 (1.646–1.892)	1.739 (1.621–1.867)
Rectum (C20)	703	0.79 (0.73–0.85)	567	1.28 (1.18–1.39)	1.622 (1.452–1.811)	1.644 (1.470–1.839)
Anus (C21)	12	0.01 (0.01–0.02)	29	0.07 (0.05–0.09)	4.856 (2.478–9.516)	4.721 (2.394–9.310)

Adjustment for sex, age, urbanization, area, insurance coverage, and co-morbidity. ^†^ Crude incidence rate per 100,000 person-years. HPV, Human Papillomavirus; HR, Hazard ratio.

## Data Availability

The data used in this research are not publicly available due to privacy and confidentiality restrictions. These data, which include patient health records and sensitive information, were accessed and analyzed under strict ethical and legal regulations. However, qualified researchers interested in accessing the data for replication or further investigation may submit requests to the National Health Insurance Administration, Ministry of Health and Welfare, Taiwan, following the established data access procedures.
